# Improving the Effect of Ferulic Acid on Inflammation and Insulin Resistance by Regulating the JNK/ERK and NF-κB Pathways in TNF-α-Treated 3T3-L1 Adipocytes

**DOI:** 10.3390/nu16020294

**Published:** 2024-01-18

**Authors:** Jae-Eun Park, Ji-Sook Han

**Affiliations:** Department of Food Science and Nutrition & Kimchi Research Institute, Pusan National University, 2 Busandaehak-ro 63 Beon-gil, Geumjeong-gu, Busan 46241, Republic of Korea; jaeeun5609@naver.com

**Keywords:** ferulic acid, inflammation, JNK/ERK, MAPK, NF-κB pathway

## Abstract

In this study, ferulic acid was investigated for its potential in suppressing TNF-α-treated inflammation and insulin resistance in adipocytes. Ferulic acid suppressed TNF-α, IL-6, IL-1β, and MCP-1. TNF-α increased p-JNK and ERK1/2, but treatment with ferulic acid (1, 10, and 50 μM) decreased p-JNK and ERK1/2. TNF-α induced the activation of IKK, IκBα, and NF-κB p65 compared to the control, but ferulic acid inhibited the activation of IKK, IκBα, and NF-κB p65. Following treatment with TNF-α, pIRS-1ser307 increased and pIRS-1tyr612 decreased compared to the control. Conversely, as a result of treatment with 1, 10, and 50 μM ferulic acid, pIRS-1ser307 was suppressed, and pIRS-1tyr612 was increased. Therefore, ferulic acid reduced inflammatory cytokine secretion by regulating JNK, ERK, and NF-κB and improved insulin resistance by suppressing pIRS-1ser. These findings indicate that ferulic acid can improve inflammation and insulin resistance in adipocytes.

## 1. Introduction

Along with insulin resistance, obesity has gained substantial attention as a causative factor of diabetes [1]. If obesity persists, inflammatory cells infiltrate the adipose tissue of the body, resulting in increased secretion of cytokines, such as tumor necrosis factor-α (TNF-α) [2]. TNF-α activates inflammation and promotes the generation of pro-inflammatory factors, including interleukin-6 (IL-6), interleukin-1 beta (IL-1β), and monocyte chemoattractant protein-1 (MCP-1) [3,4,5]. TNF-α is mainly produced in adipocytes and the peripheral tissues and induces tissue-specific inflammation, as well as insulin resistance, through the generation of ROS and activation of various transcriptional mediated pathways, especially mitogen-activated protein kinase (MAPK) pathway [6,7].

The MAPK pathway includes c-Jun *N*-terminal kinase (JNK), extracellular signal-regulated kinase (ERK), and p38 [7]. The expression of pro-inflammatory cytokines is controlled by the transcription factor activator protein 1 (AP-1) [8,9]. The activation of AP-1 is often a result of MAPK pathway regulation, particularly through the phosphorylation of c-Jun by JNK and c-Fos expression by ERK1/2 [10]. When cells are stimulated by TNF-α, the IkappaB kinase (IKK) complex becomes activated. This complex phosphorylates and ubiquitinates IκBα [11]. The NF-κB transcription factor in the cytoplasm translocates to the nucleus and initiates the transcription of various chemokines and cytokines, such as IL-6, IL-1β, and MCP-1. TNF-α triggers production of pro-inflammatory cytokines and increases IRS-1 serine phosphorylation by regulating the MAPK and NF-κB pathways, thereby inducing inflammation and insulin resistance [12].

Ferulic acid is a hydroxycinnamic acid compound contained in vegetables as well as in the seeds and cell walls of rice and oats. Ferulic acid has been demonstrated to have antioxidant, anti-inflammatory, anticancer, and antidiabetic activities [13]. However, the mechanism by which ferulic acid improves inflammation and insulin resistance in adipocytes remains unclear. In this study, we hypothesized that ferulic acid improves inflammation and insulin resistance in TNF-α-treated adipocytes. Furthermore, if ferulic acid has an improvement effect, there is a need to reveal its mechanism of action. Therefore, the objectives of this study were to investigate the effect of ferulic acid on inflammation and insulin resistance and reveal its mechanism of action in TNF-α-treated 3T3-L1 adipocytes.

## 2. Material and Methods

### 2.1. Cell Culture and Adipocyte Differentiation

The 3T3-L1 adipocytes were incubated in Dulbecco Modified Eagle Medium (DMEM, 4.5 mM glucose), with 10% Fetal Bovine Serum (FBS), at 37 °C. Cells were grown in 10% DMEM, with 1 μM dexa-methasone, 0.5 mM isobutyl-methyl-xanthine, and 10 μg/mL insulin. Thereafter, 3T3-L1 cells were incubated in DMEM with 10% FBS.

### 2.2. Enzyme-Linked Immunosorbent Assay

**Figure 1:**
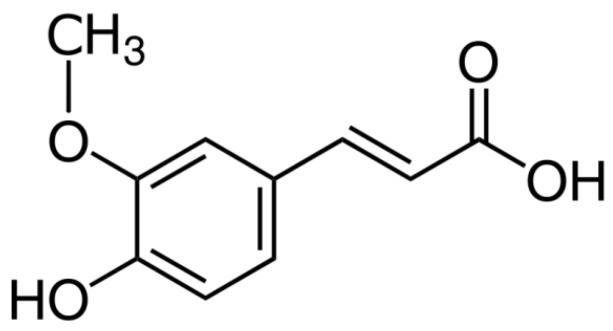
Chemical structure of ferulic acid.

Then, 3T3-L1 adipocytes and RAW 264.7 cells (2 × 104 cells/well) were cultured in 96-well plates and stimulated with TNF-α (50 ng/mL) or LPS (1 μg/mL) for 24 h. Next, they were treated with ferulic acid (1, 10, 20, and 50 μM concentrations) and incubated for another 24 h (Figure 1). After cultivation, cells were separated from the culture supernatant, and supernatant was collected. The ELISA kit (Lingo Research, Lingo, MO, USA) was used to quantify cytokines and chemokines: mouse IL-1β (ab197742), mouse IL-6 (ab222503), mouse MCP-1 (ab208979), and mouse TNF-α kit (Abcam; R&D Systems, Minneapolis, MN, USA). ELISA was performed according to the manufacturer’s protocol, as provided in each ELISA kit.

### 2.3. Cell Viability

Cell viability was evaluated using an MTT assay. In brief, cells were seeded and treated with ferulic acid for 1 day. Following treatment, MTT solution (100 µL; 1 mg/mL) was added to each well and incubated for 4 h at 37 °C. MTT-containing medium was then removed, and the resultant crystals were dissolved in dimethyl sulfoxide (100 µL) (Sigma-Aldrich, St. Louis, MO, USA). Subsequently, the optical density was measured at 540 nm using a microplate reader (Bio-Rad Laboratories Inc., Hercules, CA, USA).

### 2.4. Glucose Uptake Assay

Glucose uptake was performed by 2-[N-(7-nitrobenz-2-oxa-1, 3-diazol-4-yl)amino]-2 deoxyglucose (2-NBDG; Invitrogen, Carlsbad, CA, USA) [14]. Differentiated 3T3-L1 adipocytes at a density of 1 × 10^4^ cells/well were cultured with DMEM in 96-well plates. Inflammation and insulin resistance were induced in the cells by treating them with TNF-α (50 ng/mL) for 24 h. The cells were then treated with ferulic acid (1, 10, and 50 μM) for 24 h and exposed to insulin (100 nM) for 20 min. After removing the supernatant of the cell, 2-NBDG (10 μM) was added to each well. After 60 min, 2-NBDG uptake was investigated using a fluorescence spectrophotometer (Perkin Elmer, Waltham, MA, USA). Excitation and emission wavelengths (485 nm and 535 nm) were set. Glucose uptake was calculated using the following equation (Equation (1)):

(1) $$\mathrm{Glucose}\;\mathrm{uptake}=\frac{Control\;absorbance-sample\;absorbace}{Control\;absorbance}\times 100$$

### 2.5. Western Blot Assay

For total protein extraction from differentiated 3T3-L1 adipocytes, the cells were washed twice with ice-cold phosphate-buffered saline (PBS) and harvested in a lysis buffer (RIPA, 50 mM Tris-HCl, 150 mM NaCl, 1 mM EDTA, 1% Triton X-100, 1% sodium deoxycholate, 0.1% SDS, 1 mM phenylmethylsulfonyl fluoride (PMSF), 10 μg/mL aprotinin, 10 μg/mL leupeptin, 0.1 mM sodium orthovanadate at pH 7.4) on ice with shaking. After centrifugation at 13,000× *g* for 10 min at 4 °C, the protein content of the resulting supernatant was determined using a BCA protein assay kit (Bio-Rad Laboratories, Hercules, CA, USA). Then, 20 μg of protein was subjected to electrophoresis by 10% SDS-poly acrylamide. The separated proteins were transferred electrophoretically to nitrocellulose membrane, blocked with skim milk for 1 h, and incubated with 1st anti-bodies (JNK, ERK1/2, p38, c-Jun, c-Fos, AP-1, IKK, IκBα, NF-κB p65, IRS-1ser307, and IRStyr612; 1:1000) (Abcam, Cambridge, UK). After that, the membrane was incubated with goat anti-rabbit or goat anti-mouse 2nd anti-bodies. Complexes were visualized and detected with the luminosis analyzer LAS-1000-Plus (Fujifilm, Tokyo, Japan). Band densities were determined using an image analyzer (Multi Gauge V3.1, Fujifilm, Valhalla, NY, USA). The Band Analysis tools of Image J software version 1.8.0 were used to select and determine the bands in all the gels.

### 2.6. Statistical Analysis

Results (*n* = 3) are expressed as the mean ± standard deviation (SD). Statistical analysis was performed using SPSS version 29.0 (IBM Corp., Armonk, NY, USA). Treatment groups were compared by one-way analysis of variance (ANOVA) followed by a post hoc Duncan’s multiple-range test.

## 3. Results

### 3.1. Effect of Ferulic Acid on Inhibition of TNF-α Release in LPS-Induced RAW 264.7 Macrophages

**Figure 2:**
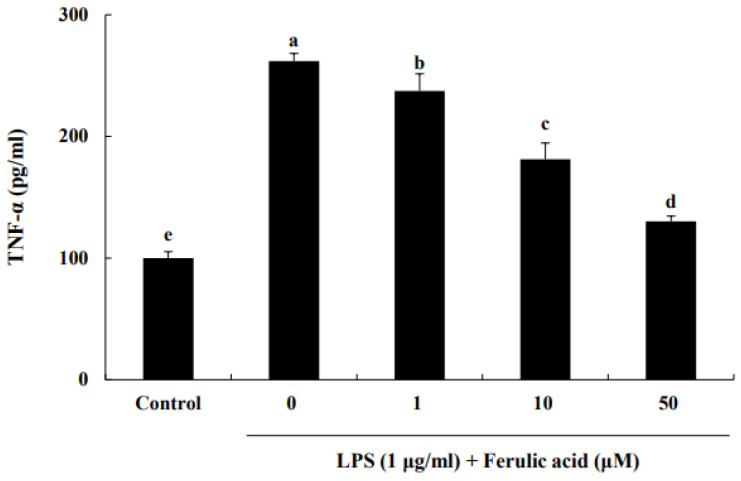
**Effect of ferulic acid on the inhibition of TNF-α release in lipopolysaccharide (LPS)-induced RAW 264.7 macrophages.** The RAW 264.7 cells were incubated with LPS (1 μg/mL) for 24 h and subsequently treated with 1, 10, and 50 μM ferulic acid for an additional 24 h. Each value is presented as the mean ± standard deviation (*n* = 3). Values with different letters (^a–e^) are considered significantly different at *p* < 0.05, as analyzed by Duncan’s multiple-range test.

TNF-α secreted from infiltrating macrophages is associated with inflammation [15]. Therefore, we investigated the TNF-α suppression effect of ferulic acid in LPS-induced RAW macrophages. LPS increased TNF-α release, leading to a significant increase in TNF-α levels by 261.81% compared to those of the control (Figure 2). But treatment with 1, 10, and 50 μM ferulic acid reduced TNF-α levels by 237.36%, 181.19%, and 130.26%, respectively.

### 3.2. Effect of Ferulic Acid on Cell Viability and Reduction in Cytokines and Chemokines in TNF-α-Treated Adipocytes

**Figure 3:**
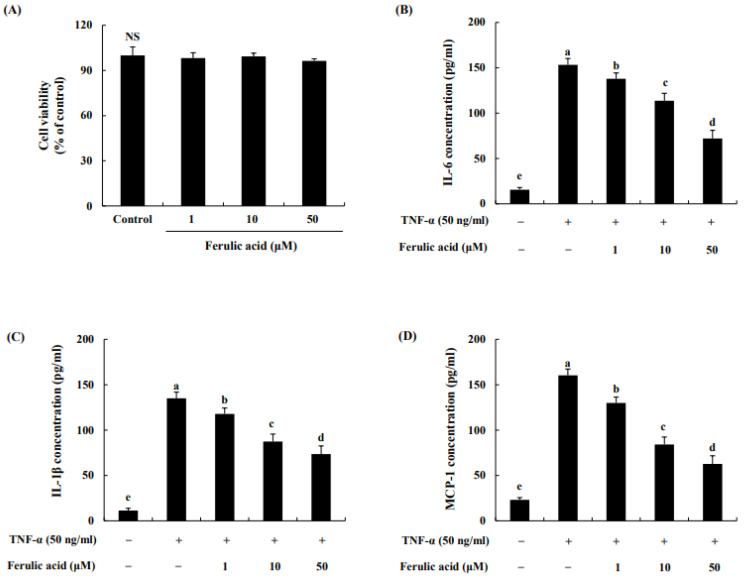
**Effect of ferulic acid on cell viability and the reduction in cytokines and chemokines in TNF-α-treated adipocytes.** (**A**) Cytotoxic effects of HM-chromanone. (**B**) Production of interleukin (IL)-6. (**C**) Production of IL-1β. (**D**) Production of monocyte chemoattractant protein (MCP)-1. Each value is presented as the mean ± standard deviation (*n* = 3). Values with different letters (^a–e^) are significantly different at *p* < 0.05, as analyzed by Duncan’s multiple-range test. NS: Not Significant.

The effect of ferulic acid on the cell viability of adipocytes was assessed using the MTT assay. We found that 1, 10, or 50 μM ferulic acid treatment did not affect cell viability (Figure 3A). Thus, the possibility of cellular toxicity contributing to the anti-inflammatory effect of ferulic acid on adipocytes was excluded. The levels of cytokines and chemokines are shown in Figure 3B–D. IL-6 levels were increased by treatment with TNF-α (153.29 pg/mL) compared to the control (14.49 pg/mL) (Figure 3B). When treated with 1, 10, or 50 μM ferulic acid and TNF-α, IL-6 levels were decreased to 137.81, 113.64, and 72.02 pg/mL, respectively. Similarly, IL-1β levels were increased by the treatment of TNF-a (134.97 pg/mL) compared to the control (11.29 pg/mL) (Figure 3C). When treated with 1, 10, or 50 μM ferulic acid and TNF-α, the levels of IL-1β were significantly decreased to 117.35, 87.32, and 73.59 pg/mL, respectively. MCP-1 levels were increased by TNF-α (160.37 pg/mL) compared to the control (23.10 pg/mL) (Figure 3D). However, when treated with 1, 10, or 50 μM ferulic acid and TNF-α, MCP-1 levels were significantly decreased to 129.88, 84.04, and 62.60 pg/mL, respectively.

### 3.3. Effect of Ferulic Acid on the MAPK Pathway in TNF-α-Treated Adipocytes

**Figure 4:**
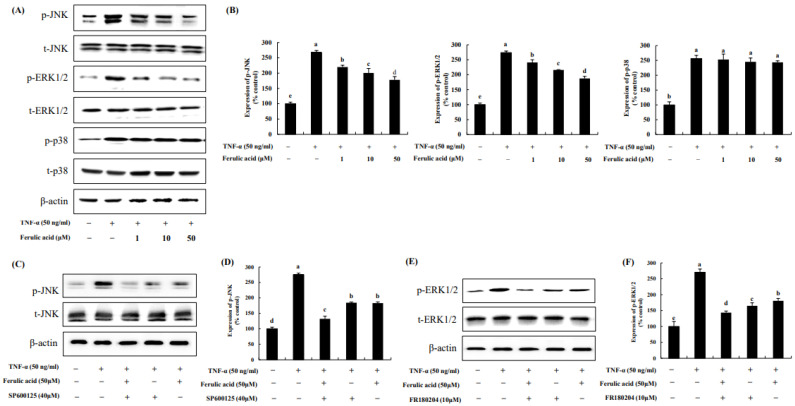
**Effect of ferulic acid on the MAPK pathway in TNF-α-treated adipocytes.** (**A**) Expression of phosphorylated JNK, pERK1/2, and p38 was determined using Western blotting. (**B**) Expression values of pJNK, pERK1/2, and p38. (**C**) Expression of pJNK was assessed with SP600125 (40 μM, JNK inhibitor). (**D**) Expression values of pJNK with SP600125 treatment. (**E**) Expression of pERK1/2 was examined with FR180204 (10 μM, ERK1/2 inhibitor). (**F**) Expression values of pERK1/2 with FR180204 treatment. β-actin was utilized as the loading control. Each value is presented as the mean ± standard deviation (*n* = 3). Values with different letters (^a–e^) are significantly different at *p* < 0.05, as analyzed by Duncan’s multiple-range test.

The data show that TNF-α increased p- JNK, ERK1/2, and p38 by 267.38%, 273.80%, and 256.27%, respectively, compared to the control (100%) (Figure 4A,B). In contrast, ferulic acid treatment dose-dependently decreased p-JNK and ERK1/2. p- JNK and ERK1/2 were inhibited by 220.04%, 200.43%, and 177.94 and 241.11%, 215.90%, and 187.36% with 1, 10, and 50 μM ferulic acid, respectively. However, it did not affect the phosphorylation of p38. These effects were confirmed using JNK and ERK1/2 inhibitors. The JNK inhibitor, SP600125, inhibited JNK phosphorylation in TNF-α-treated cells (Figure 4C,D), while the ERK1/2 inhibitor, FR180204, inhibited ERK1/2 phosphorylation under TNF-α treatment (Figure 4E,F). Moreover, treatment with ferulic acid and SP600125 or FR180204 inhibited TNF-α-treated JNK and ERK1/2 more than treatment with the JNK or ERK inhibitor alone.

### 3.4. Effect of Ferulic Acid on the Expression of c-Jun, c-Fos, and AP-1 in TNF-α-Treated Adipocytes

**Figure 5:**
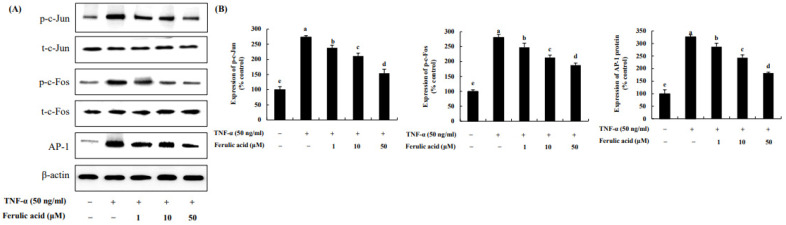
**Effect of ferulic acid on the expression of c-Jun, c-Fos, and AP-1 in TNF-α-treated adipocytes**. (**A**) Expressions of c-Jun, c-Fos, and AP-1 were determined using Western blotting. (**B**) Expression values of c-Jun, c-Fos, and AP-1. β-actin was used as the loading control. Each value is presented as the mean ± standard deviation (*n* = 3). Values with different letters (^a–e^) are significantly different at *p* < 0.05, as analyzed by Duncan’s multiple-range test.

We evaluated the effects of ferulic acid on c-Jun, c-Fos, and AP-1 in TNF-α-treated adipocytes (Figure 5). C-Jun and c-Fos, due to TNF-α, were increased by 273.49% and 281.04%, respectively, compared to the control (100%). However, treatment with 1, 10, and 50 μM ferulic acid in combination with TNF-α reduced the phosphorylation of c-Jun (by 238.16%, 211.07%, and 154.42%, respectively) and c-Fos (by 247.78%, 213.65%, and 188.12%, respectively). Additionally, AP-1 was elevated by 326.17% by TNF-α compared to the control, but it was inhibited by 287.08%, 243.33%, and 182.15% with 1, 10, and 50 μM ferulic acid.

### 3.5. Effect of Ferulic Acid on the NF-κB Pathway in TNF-α-Treated Adipocytes

**Figure 6:**
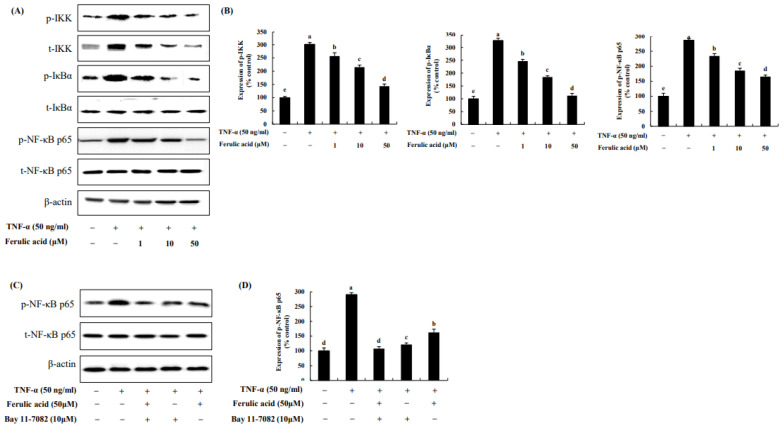
**Effect of ferulic acid on the NF-κB pathway in TNF-α-treated adipocytes.** (**A**) Expression of inhibitor kappa B (IκB) kinase (IKK), IκBα, and NF-κB/p65 was determined using Western blotting. (**B**) Expression values of IKK, IκBα, and NF-κB/p65. (**C**) Expression of pNF-κB/p65 was examined with Bay11-7082 (10 μM, NF-κB/p65 inhibitor). (**D**) Expression values of pNF-κB/p65 with Bay11-7082 treatment. β-actin was utilized as the loading control. Each value is presented as the mean ± standard deviation (*n* = 3). Values with different letters (^a–e^) are significantly different at *p* < 0.05, as analyzed by Duncan’s multiple-range test.

IKK, IκBα, and NF-κB p65 were increased by 301.34%, 326.77%, and 286.20%, respectively, upon TNF-α treatment compared to the control (100%) (Figure 6). However, treatment with ferulic acid decreased the phosphorylation of IKK, IκBα, and NF-κBp65. Treatment with both ferulic acid and Bay 11-7082 (NF-κB inhibitor) inhibited NF-κB phosphorylation to a greater extent than treatment with the NF-κB inhibitor alone in TNF-α-treated adipocytes, indicating that ferulic acid inhibits p-NF-κB (Figure 6C,D).

### 3.6. Effect of Ferulic Acid on the Phosphorylation of IRS-1 Residues in TNF-α-Treated Adipocytes

**Figure 7:**
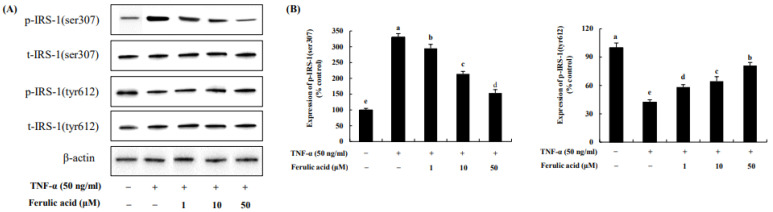
**Effect of ferulic acid on the phosphorylation of IRS-1 residues in TNF-α-treated adipocytes.** (**A**) Expressions of IRS-1serine (ser)307 and IRS-1tyrosine (tyr)612 were determined using Western blotting. (**B**) Expression values of IRS-1 (ser307) and IRS-1 (tyr612). β-actin was used as the loading control. Each value is expressed as mean ± standard deviation (*n* = 3). ^a–e^ Values with other letters are significantly different at *p* < 0.05, as analyzed by Duncan’s multiple-range test.

The effect of ferulic acid on p-IRS-1 residues in TNF-α-treated adipocytes was investigated (Figure 7). The phosphorylation of IRS-1serine 307, induced by TNF-a, was increased by 330.83%. However, ferulic acid (10, 20, 50 μM) treatment significantly decreased p-IRS-1ser (295.06%, 214.27%, and 153.49%, respectively). But the phosphorylation of IRS-1 tyrosine 612, following TNF-α, was decreased by 42.37% compared to the control. However, ferulic acid treatment significantly increased p- IRS-1tyr (10, 20, 50 μM: 58.29%, 64.54%, and 81.1%, respectively). This indicates that ferulic acid inhibits IRS-1ser307 phosphorylation and increases IRS-1tyr612 phosphorylation in TNF-α-treated adipocytes.

### 3.7. Effect of Ferulic Acid on Insulin-Stimulated Glucose Uptake in TNF-α-Treated Adipocytes

**Figure 8:**
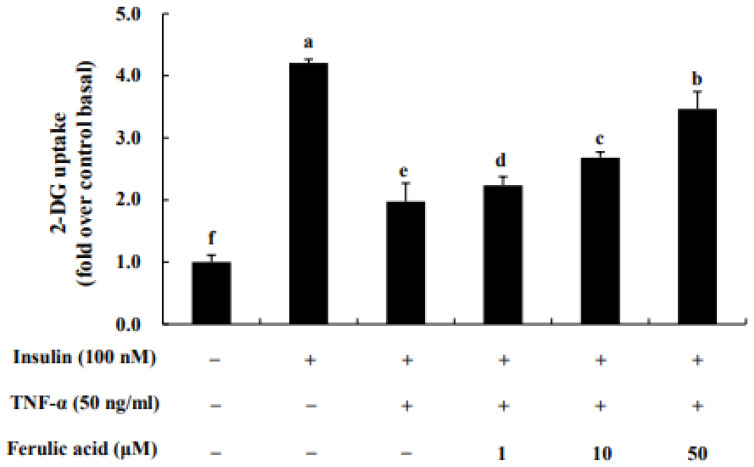
**Effect of ferulic acid on insulin-stimulated glucose uptake in TNF-α-treated adipocytes.** Differentiated 3T3-L1 adipocytes at a density of 1 × 104 cells/well were cultured with DMEM in 96-well plates. Inflammation and insulin resistance were induced in the cells by treating them with TNF-α (50 ng/mL) for 24 h. The cells were then treated with ferulic acid (1, 10, and 50 μM) for 24 h and exposed to insulin (100 nM) for 20 min. Each value is expressed as the mean ± standard deviation (*n* = 3). ^a–f^ Values with other letters are significantly different at *p* < 0.05, as analyzed by Duncan’s multiple-range test.

Uptake of 2-DG glucose was assessed following treatment of TNF-α in adipocytes (Figure 8). When cells were treated with TNF-α, glucose uptake decreased 1.97-fold. However, glucose uptake was restored concentration-dependently with ferulic acid treatment. Treatment with 1, 10, and 50 μM ferulic acid of TNF-α significantly increased glucose uptake (by 2.23-fold, 2.68-fold, and 3.46-fold, respectively).

## 4. Discussion

Infiltrated macrophages release TNF-α, which can induce insulin resistance in adipocytes [16]. According to our preliminary experiment, ferulic acid (1 to 50 μM) significantly inhibits TNF-α production in LPS-treated RAW cells. In addition, treatment with ferulic acid (50 μM) showed upregulation of antioxidant defenses and suppression of inflammatory events by inhibiting NFκB activation [17]. Thus, the 1 μM and 50 μm treatments, which show an anti-inflammatory effect, were selected and used for the experiments. Our results indicate that ferulic acid (1–50 μM), a type of phenolic acid, dose-dependently inhibited TNF-α production in LPS-treated RAW cells. According to the findings of Kwon et al., caffeic acid inhibits LPS-mediated TNF-α production in RAW cells [18]. In addition, various phenolic compounds, such as quercetin and luteolin, which contain hydroxyl groups, have been observed to exhibit stronger effects than non-hydroxyl-containing compounds on the inhibition of TNF-α production in RAW cells [19]. The inhibition of TNF-α production by phenolic compounds is thought to be closely related to their chemical structures. Ferulic acid possesses one hydroxyl group and one methoxy group in its structure [19]. The structure of ferulic acid may partially contribute to reducing TNF-α production secreted from macrophages.

TNF-α plays a crucial role in inflammation. It triggers the inflammatory signaling pathway in adipocytes, leading to the generation of inflammatory cytokines and chemokines like IL-1β, IL-6, and MCP-1 [20]. Recent studies have identified IL-1β as a potential contributor to the development of insulin resistance and type 2 diabetes [21]. Inhibition of IL-1β has been shown to reduce hyperglycemia and inflammation in obese mice and diabetic rats [22]. Furthermore, among the pro-inflammatory cytokines, IL-6 emerged as one of the potential mediators that links obesity-derived chronic inflammation to insulin resistance [23,24]. Its expression can be induced by other inflammatory cytokines, such as IL-1β. As with TNFα, the plasma level of IL-6 increases with obesity and insulin resistance [25]. Elevated levels of IL-6 and IL-1β have been shown to increase the risk of type 2 diabetes [26]. MCP-1 is one of the key chemokines that promotes direct macrophage infiltration into adipose tissue [27]. Increased MCP-1 expression in adipose tissue contributes to chronic inflammation and insulin resistance [28].

In our study, TNF-a treatment similarly increased the levels of IL-1β, IL-6, and MCP-1 in adipocytes. However, treatment with ferulic acid effectively suppressed the production of cytokines and chemokines in TNF-α-treated adipocytes. According to one study, catechin, a natural polyphenolic compound, can attenuate the inflammatory response triggered by TNF-a through the inhibition of IL-1β and IL-6 in 3T3-L1 adipocytes [29]. In addition, piceatannol, a naturally occurring polyphenolic compound, considerably suppressed the release of TNF-α and the secretion of IL-6, IL-1β, and MCP-1, thereby attenuating pathological inflammation in adipose tissue [30,31]. These results suggest that ferulic acid might alleviate inflammation by suppressing the production of IL-6, IL-1β, and MCP-1 in TNF-α-treated adipocytes.

MAPKs comprise three major members: JNK, ERK1/2, and p38 [32,33,34,35]. Our study showed that TNF-α increased the phosphorylation of p-JNK, ERK1/2, and p38. Conversely, ferulic acid reduced JNK and ERK1/2 phosphorylation, but no significant change was observed in p38 phosphorylation. This was confirmed by using inhibitors of the MAPK pathway. The JNK inhibitor SP600125 inhibited JNK phosphorylation in TNF-α-treated cells, whereas the ERK1/2 inhibitor FR180204 inhibited ERK1/2 phosphorylation under TNF-α treatment. In addition, treatment with ferulic acid and SP600125 or FR180204 inhibited TNF-α-induced JNK and ERK1/2 phosphorylation to a greater extent than treatment with the JNK or ERK inhibitor alone. Additionally, c-Jun phosphorylation was inhibited through the reduction in JNK phosphorylation; c-Fos phosphorylation was inhibited through the reduction in ERK1/2 phosphorylation, and AP-1 expression was suppressed through these pathways. Fernanda et al. (2021) demonstrated that hydroxycinnamic acid derivatives, such as coumaric acid, possessed strong anti-inflammation effects. Additionally, hydroxycinnamic acid derivatives also inhibited JNK and ERK1/2, as well as c-Fos, consequently inhibiting the expression of AP-1 [36,37,38]. According to one study, the removal of the hydroxyl group resulted in the loss of the JNK inhibitory effect in phenolic compounds [39,40]. Therefore, it can be assumed that the hydroxyl group contributes to the ability of ferulic acid to reduce JNK activation and AP-1 expression.

In general, the NF-κB complex exists in an inactive state in the cytoplasm. When stimulated by molecules such as TNFα, or other cell stressors, TNFα binds to TNF receptors. This binding, via several intermediate steps, leads to an interaction with the IκB kinase (IKK) complex, which then leads to the phosphorylation of IκB, and subsequently results in IκB ubiquitination and degradation [41]. After being degraded by the proteasome, NF-κB p65 is released and translocated into the nucleus, inducing the expression of the inflammatory cytokines MCP-1 and IL-6, causing an inflammatory response [42,43]. Our study demonstrated that ferulic acid inhibited IKK and IκBα phosphorylation as well as NF-κB activation. Quercetin substantially inhibited IKK and IκBα phosphorylation and reduced NF-κB and AP-1 activity in TNF-induced adipocytes [44,45]. Treatment with luteolin, a type of flavonoid, in SW982 cells has been found to substantially decrease TNF-α and IL-6 production, inhibit MAPKs (JNK and p38), and activate NF-κB transcription factors. Collectively, these results suggest that ferulic acid can inhibit TNF-α-induced inflammation by regulating the IKK/NF-κB signaling pathway in adipocytes.

JNK and IKK activated by TNF-α promote the serine phosphorylation of IRS-1, ultimately impairing insulin action and downregulating glucose uptake in adipocytes [46,47,48]. However, ferulic acid reversed the TNF-α-induced increase in IRS-1 serine phosphorylation and decrease in IRS-1 tyrosine phosphorylation by inhibiting JNK and IKK. In addition, ferulic acid increased glucose uptake in 3T3-L1 adipocytes. Although our study provided novel insights into the effects of ferulic acid on improvement in inflammation and insulin resistance by regulating the JNK/ERK and NF-κB pathways in TNF-α-treated adipocytes, the results obtained should be evaluated in an animal model, and finally, in a clinical trial.

## 5. Conclusions

**Figure 9:**
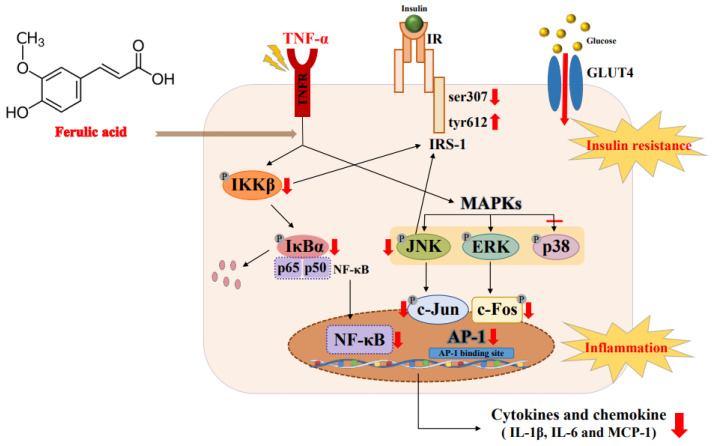
Mechanism by which ferulic acid improves inflammation and insulin resistance via regulating the JNK/ERK and NF-κB pathways in TNF-α-treated 3T3-L1 adipocytes. The explanation of the red arrow: ↑ increase, ↓ decrease, – suppression.

TNF-a triggered the JNK/ERK and NF-κB signaling pathways. But ferulic acid inhibited the secretion of the inflammatory cytokines TNF-α, IL-6, and IL-1β, along with the chemokine MCP-1. Ferulic acid suppressed JNK/ERK1/2, blocked IκBα phosphorylation, and inhibited NF-κB. Additionally, ferulic acid reduced pIRS-1ser, while increasing tyrosine 612 phosphorylation of IRS-1, thus restoring glucose uptake. These results suggest that ferulic acid could ameliorate inflammation and insulin resistance by regulating the JNK/ERK and NF-κB pathways in TNF-α-treated adipocytes (Figure 9).

## Data Availability

All the data is contained in the manuscript.

## References

[1] Lin X., Li H. (2021). Obesity: Epidemiology, Pathophysiology, and Therapeutics. Front. Endocrinol..

[2] Chait A., Hartigh L.J. (2020). Adipose Tissue Distribution, Inflammation and Its Metabolic Consequences, Including Diabetes and Cardiovascular Disease. Front. Cardiovasc. Med..

[3] Khanna D., Khanna S., Khanna P., Kahar P., Patel B.M. (2022). Obesity: A Chronic Low-Grade Inflammation and Its Markers. Cureus.

[4] Kojta I., Chacińska M., Błachnio-Zabielska A. (2020). Obesity, Bioactive Lipids, and Adipose Tissue Inflammation in Insulin Resistance. Nutrients.

[5] Kawai T., Autieri M.V., Scalia R. (2021). Adipose tissue inflammation and metabolic dysfunction in obesity. Am. J. Physiol. Cell Physiol..

[6] Akash M.S.H., Rehman K., Liaqat A. (2018). Tumor Necrosis Factor-Alpha: Role in Development of Insulin Resistance and Pathogenesis of Type 2 Diabetes Mellitus. J. Cell Biochem..

[7] Pearson G., Robinson F., Beers Gibson T. (2001). Mitogen-activated protein (MAP) kinase pathways: Regulation and physiological functions. Endocr. Rev..

[8] Johnson G.L., Lapadat R. (2002). Mitogen-activated protein kinase pathways mediated by ERK, JNK, and p38 protein kinases. Science.

[9] Kyriakis J.M. (1999). Activation of the AP-1 transcription factor by inflammatory cytokines of the TNF family. Gene Expr..

[10] Yue J., López J.M. (2020). Understanding MAPK Signaling Pathways in Apoptosis. Int. J. Mol. Sci..

[11] Israël A. (2010). The IKK complex, a central regulator of NF-kappaB activation. Cold Spring Harb. Perspect. Biol..

[12] Rui L., Aguirre V., Kim J.K. (2001). Insulin/IGF-1 and TNF-alpha stimulate phosphorylation of IRS-1 at inhibitory Ser307 via distinct pathways. J. Clin. Investig..

[13] Kumar N., Pruthi V. (2014). Potential applications of ferulic acid from natural sources. Biotechnol. Rep..

[14] Alonso-Castro A.J., Miranda-Torres A.C., González-Chávez M.M., Salazar-Olivo L.A. (2008). Cecropia obtusifolia Bertol and its active compound, chlorogenic acid, stimulate 2-NBDglucose uptake in both insulin-sensitive and insulin-resistant 3T3 adipocytes. J. Ethnopharmacol..

[15] Aladhami A.K., Unger C.A., Ennis S.L., Altomare D., Ji H., Hope M.C., Velázquez K.T., Enos R.T. (2021). Macrophage tumor necrosis factor-alpha deletion does not protect against obesity-associated metabolic dysfunction. FASEB J..

[16] Xu H., Barnes G.T., Yang Q., Tan G., Yang D., Chou C.J., Sole J., Nichols A., Ross J.S., Tartaglia L.A. (2003). Chronic inflammation in fat plays a crucial role in the development of obesity-related insulin resistance. J. Clin. Investig..

[17] Mir S.M., Ravuri H.G., Pradhan R.K., Narra S., Kumar J.M., Kuncha M., Kanjilal S., Sistla R. (2018). Ferulic acid protects lipopolysaccharide-induced acute kidney injury by suppressing inflammatory events and upregulating antioxidant defenses in Balb/c mice. Biomed. Pharmacother..

[18] Kwon M.Y., Kim S.M., Park J. (2019). A caffeic acid-ferulic acid hybrid compound attenuates lipopolysaccharide-mediated inflammation in BV2 and RAW264.7 cells. Biochem. Biophys. Res. Commun..

[19] Comalada M., Ballester I., Bailón E. (2006). Inhibition of pro-inflammatory markers in primary bone marrow-derived mouse macrophages by naturally occurring flavonoids: Analysis of the structure-activity relationship. Biochem. Pharmacol..

[20] Suganami T., Nishida J., Ogawa Y. (2005). A paracrine loop between adipocytes and macrophages aggravates inflammatory changes: Role of free fatty acids and tumor necrosis factor alpha. Arterioscler. Thromb. Vasc. Biol..

[21] Tack C.J., Stienstra R., Joosten L.A., Netea M.G. (2012). Inflammation links excess fat to insulin resistance: The role of the interleukin-1 family. Immunol. Rev..

[22] Sauter N.S., Schulthess F.T., Galasso R., Castellani L.W., Maedler K. (2008). The antiinflammatory cytokine interleukin-1 receptor antagonist protects from high-fat diet-induced hyperglycemia. Endocrinology.

[23] Kim J.H., Bachmann R.A., Chen J. (2009). Interleukin-6 and insulin resistance. Vitam. Horm..

[24] Almuraikhy S., Kafienah W., Bashah M. (2016). Interleukin-6 induces impairment in human subcutaneous adipogenesis in obesity-associated insulin resistance. Diabetologia.

[25] Popko K., Gorska E., Stelmaszczyk-Emmel A. (2010). Proinflammatory cytokines Il-6 and TNF-α and the development of inflammation in obese subjects. Eur. J. Med. Res..

[26] Spranger J., Kroke A., Möhlig M. (2003). Inflammatory cytokines and the risk to develop type 2 diabetes: Results of the prospective population-based European Prospective Investigation into Cancer and Nutrition (EPIC)-Potsdam Study. Diabetes.

[27] Deshmane S.L., Kremlev S., Amini S., Sawaya B.E. (2009). Monocyte chemoattractant protein-1 (MCP-1): An overview. J. Interferon. Cytokine Res..

[28] Kanda H., Tateya S., Tamori Y., Kotani K., Hiasa K., Kitazawa R., Kitazawa S., Miyachi H., Maeda S., Egashira K. (2006). MCP-1 contributes to macrophage infiltration into adipose tissue, insulin resistance, and hepatic steatosis in obesity. J. Clin. Investig..

[29] Cheng A.W., Tan X., Sun J.Y., Gu C.M., Liu C., Guo X. (2019). Catechin attenuates TNF-α induced inflammatory response via AMPK-SIRT1 pathway in 3T3-L1 adipocytes. PLoS ONE.

[30] Yamamoto T., Li Y., Hanafusa Y. (2016). Piceatannol exhibits anti-inflammatory effects on macrophages interacting with adipocytes. Food Sci. Nutr..

[31] Kershaw J., Kim K.H. (2017). The Therapeutic Potential of Piceatannol, a Natural Stilbene, in Metabolic Diseases: A Review. J. Med. Food..

[32] Zhao L., Liu X., Liang J., Han S., Wang Y., Yin Y. (2013). Phosphorylation of p38 MAPK mediates hypoxic preconditioning-induced neuroprotection against cerebral ischemic injury via mitochondria translocation of Bcl-xL in mice. Brain Res..

[33] Lennmyr F., Ericsson A., Gerwins P., Ahlstrom H., Terent A. (2003). Increased brain injury and vascular leakage after pretreatment with p38-inhibitor SB203580 in transient ischemia. Acta Neurol. Scand..

[34] Karin M., Liu z.g., Zandi E. (1997). AP-1 function and regulation. Curr. Opin. Cell Biol..

[35] Ventura J.J., Kennedy N.J., Lamb J.A., Flavell R.A., Davis R.J. (2003). c-Jun NH(2)-terminal kinase is essential for the regulation of AP-1 by tumor necrosis factor. Mol. Cell Biol..

[36] Park S.H., Ko J.W., Shin N.R. (2017). 4-Hydroxycinnamic acid protects mice from cigarette smoke-induced pulmonary inflammation via MAPK pathways. Food Chem. Toxicol..

[37] Aquino F.L.T., Silva J.P.D., Ferro J.N.S., Lagente V., Barreto E. (2021). trans-Cinnamic acid, but not p-coumaric acid or methyl cinnamate, induces fibroblast migration through PKA- and p38-MAPK signalling pathways. J. Tissue Viability.

[38] Hseu Y.C., Korivi M., Lin F.Y. (2018). Trans-cinnamic acid attenuates UVA-induced photoaging through inhibition of AP-1 activation and induction of Nrf2-mediated antioxidant genes in human skin fibroblasts. J. Dermatol. Sci..

[39] Lin C.W., Hou W.C., Shen S.C. (2008). Quercetin inhibition of tumor invasion via suppressing PKC delta/ERK/AP-1-dependent matrix metalloproteinase-9 activation in breast carcinoma cells. Carcinogenesis.

[40] Li X.J., Zhu Z., Han S.L., Zhang Z.L. (2016). Bergapten exerts inhibitory effects on diabetes-related osteoporosis via the regulation of the PI3K/AKT, JNK/MAPK and NF-κB signaling pathways in osteoprotegerin knockout mice. Int. J. Mol. Med..

[41] Gloire G., Legrand-Poels S., Piette J. (2006). NF-kappaB activation by reactive oxygen species: Fifteen years later. Biochem. Pharmacol..

[42] Chen Y., Yang L., Lee T.J. (2000). Oroxylin A inhibition of lipopolysaccharide-induced iNOS and COX-2 gene expression via suppression of nuclear factor-κB activation. Biochem. Pharmacol..

[43] Kim J.W., Kim C. (2005). Inhibition of LPS-induced NO production by taurine chloramine in macrophages is mediated though Ras-ERK-NF-κB. Biochem. Pharmacol..

[44] Chang Y.C., Tsai M.H., Sheu W.H., Hsieh S.C., Chiang A.N. (2013). The therapeutic potential and mechanisms of action of quercetin in relation to lipopolysaccharide-induced sepsis in vitro and in vivo. PLoS ONE.

[45] Zoico E., Nori N., Darra E. (2021). Senolytic effects of quercetin in an in vitro model of pre-adipocytes and adipocytes induced senescence. Sci. Rep..

[46] Liu T., Zhang L., Joo D., Sun S.C. (2017). NF-κB signaling in inflammation. Signal. Transduct. Target Ther..

[47] Langlais P., Yi Z., Finlayson J. (2011). Global IRS-1 phosphorylation analysis in insulin resistance. Diabetologia.

[48] Yung J.H.M., Giacca A. (2020). Role of c-Jun N-terminal Kinase (JNK) in Obesity and Type 2 Diabetes. Cells.

